# Non-coding RNA-related FCGBP downregulation in head and neck squamous cell carcinoma: a novel biomarker for predicting paclitaxel resistance and immunosuppressive microenvironment

**DOI:** 10.1038/s41598-024-55210-6

**Published:** 2024-02-23

**Authors:** Qin Ding, Fengjie Lin, Zongwei Huang, Ying Li, Sunqin Cai, Xin Chen, Hui Liu, Sufang Qiu

**Affiliations:** 1 Department of Radiation Oncology, Clinical Oncology School of Fujian Medical University, Fujian Cancer Hospital, Fuzhou, 350014 China; 2 Fujian Provincial Key Laboratory of Translational Cancer Medicine, Fuzhou, China

**Keywords:** Fc gamma binding protein, Paclitaxel resistance, Immune infiltration, Targeted therapy, Head and neck cancer, Biochemistry, Immunochemistry, Proteomics, RNA

## Abstract

In head and neck squamous cell carcinoma (HNSC), chemoresistance is a major reason for poor prognosis. Nevertheless, there is a lack of validated biomarkers to screen for patients for categorical chemotherapy. Fc gamma binding protein (FCGBP) is a mucus protein associated with mucosal epithelial cells and has immunological functions that protect against tumors and metastasis. However, the effect of FCGBP on HNSC is unclear. In pan-cancer tissues, the expression of FCGBP and the survival status of patients were analyzed using information from The Cancer Genome Atlas (TCGA) and Gene Expression Omnibus (GEO). Correlation analysis and Cox regression analysis were conducted to confirm the relationship and survival outcome. Bioinformatics analysis was utilized to predict the probable upstream non-coding RNA. FCGBP functioned as a potential tumor suppressor gene in HNSC. Notably, FCGBP expression was negatively correlated with enriched tumor-infiltrating macrophages and paclitaxel resistance. Cox regression with gene, clinical, and immune factors showed that FCGBP was a risk factor acting in an independent manner. In HNSC, the utmost possibly upstream non-coding RNA-related pathway of FCGBP was also discovered to be the PART1/AC007728.2/LINC00885/hsa-miR-877-5p/FCGBP axis. According to the present study, non-coding RNA-related low levels of FCGBP are a prognostic indicator and are linked to an HNSC-related immunosuppressive state.

## Introduction

As the seventh most common prevalent cancer worldwide, head and neck cancers are mainly composed of squamous cell carcinomas^1,2^. Most (> 50%) of head and neck squamous cell carcinoma (HNSC) show a local progression or distant metastasis^3^. Irrespective of multiple therapeutic approaches, the results for advanced HNSC patients’ are not encouraging; with 40–60% of patients experiencing local progression or recurrence and a poor prognosis^4^. The resistance to treatment, particularly to chemotherapy, is an overwhelming factor in poor prognosis^1,5^. Thus, for stratified chemotherapy and prognosis, there is an urgent need to identify viable biomarkers as well as new therapeutic targets for effectively managing HNSC.

A mucin called Fc gamma binding protein (FCGBP) was first discovered in the intestinal epithelium and encodes a cysteine-rich glycoprotein^6^. It is a key factor in the protection of innate mucosal epithelium, tumor immunity, and tumor metastasis^7^. The expression of FCGBP varies across tissues, which has been linked to tumorigenesis in many malignancies^8–14^. Besides, the FCGBP level was one of the top differentially expressed transcripts between chemosensitive and chemoresistant tissues^15^.

However, few reports have been dedicated to the chemotherapeutic effects of FCGBP on the tumor. Lin YH et al. reported FCGBP as a potential biomarker to predict HNSC prognosis^16^. These findings prompted us to delve into the expression, prognosis, and possible mechanisms of FCGBP in HNSC. Furthermore, even though FCGBP is confirmed to have a positive link to immune cells, the association of FCGBP with drug resistance as well as the inhibitory immune microenvironment in HNSC is still unclear.

The FCGBP expression and prognostic significance in tumor tissue were investigated in this research work. Moreover, the impact of FCGBP on susceptibility to drugs was first studied. Notably, we provided an in-depth study on the roles of FCGBP in HNSC immune infiltration. In addition, non-coding RNAs (ncRNAs), as well as microRNAs (miRNAs) associated with FCGBP regulation were also performed mechanistically in HNSC. In conclusion, our findings demonstrated that the ncRNA-related downregulation of FCGBP was involved in bad prognosis, paclitaxel resistance, and the immune-suppressive microenvironment in HNSC.

## Materials and methods

### Data pre-processing

The UCSC (https://xenabrowser.net/) database was searched to download the normalized pan-cancer dataset, TCGA Pan-Cancer (PANCAN, N = 10,535, G = 60,499). Expression information for ENSG00000275395 (FCGBP) was taken separately from each sample and analyzed using the log2 (x + 1) method. UCSC (https://xenabrowser.net/) was also employed to acquire transcriptional data and clinicopathological features of HNSC patients (n = 546). The gene expression of GSE42743, GSE75538, GSE84713 were obtained from Gene Expression Omnibus (GEO) database. The “limma” package was used for normalization. We performed an immunohistochemical analysis utilizing the Human Protein Atlas (HPA, https://www.proteinatlas.org/). Univariate and multivariate Cox regression analysis of HNSC patient survival were performed as well as CIBERSORTx methods.

### Immunohistochemistry analysis

HNSC biopsies were fixed with 10% formalin overnight and processed into 5-μm-thick paraffin sections. The slides were then analyzed by immunohistochemistry with anti-human FCGBP (HPA003564; Merck) followed by HRP secondary antibody (Cat #ab205718; Abcam) and DAB staining. Images were obtained using a microscope (BX43; Olympus, Japan). Histochemistry score (H-score) was used to evaluate the expression. H-score = ∑ (pi × i) = (percentage of weak intensity × 1) + (percentage of moderate intensity × 2) + (percentage of strong intensity × 3).

### Differential expression and survival analysis

Tumor Immune Estimation Resource (TIMER) is an extensive source of data that can be tested on tumor and normal samples in a wide range of cancer studies. Utilizing the Cox model, the correlation between each cancer’s prognosis and gene expression was investigated.

The log-rank test was employed for the comparison across two survival curves. Further validation of these findings was accomplished utilizing the UALCAN and Gene Expression Profiling Interactive Analysis (GEPIA) databases. The Cox test was employed to do a survival analysis for FCGBP expression and candidate lncRNAs in cancers. The survival of CGBP and candidate lncRNAs were investigated using the Kaplan–Meier test.

### CIBERSORTx and macrophages analysis of tumour immunology estimation resources

Utilizing the R package “ESTIMATE”, we estimated the ESTIMATE scores. Then, to evaluate the gene expression of single samples, we employed a single-sample gene set enrichment analysis (ssGSEA) technique. The 22 subtypes of immune cells from TCGA were then counted using the CIBERSORTx algorithm. In this study, 10 types of immune cells, including B cells, dendritic cells, CD8 T cells, CD4 T cells, γ–δ T cells, natural killer cells, plasma cells, macrophages (M0 macrophages, M1 macrophages, M2 macrophages), monocytes, and neutrophils, were analyzed. Moreover, we employed multiple datasets (TCGA-HNSC, GSE42743, GSE75538, GSE84713) to generate immune estimates, providing additional validation and scrutiny of the CIBERSORT outcomes. Given the current state of our study, it is important to emphasize that propositions about immune cell infiltration are based entirely on speculation about mRNA expression. Additionally, a total of 10 stimulated immune response-related genes were obtained from the molecular signatures database (MSigDB) by searching for “stimulated immune response”. From a preview study, nine immune checkpoints were identified whose effectiveness had been demonstrated^17^.

### Correlation of prognosis with immune cells and clinicopathological factors in FCGBP sub-group

Findings from a univariate Cox regression analysis were utilized to examine the factors influencing overall survival (OS) in HNSC patients. Two different levels of immune cell infiltration, high and low, were measured separately by intermediate values. The univariate analysis considered all parameters, initially with a threshold of *p* < 0.05, and subsequently, the multivariate model incorporated only those with *p* < 0.1 of univariate analysis.

### Drug sensitivity analysis

To unravel the functional implications of FCGBP in drug sensitivity and tolerance, we embarked on a sophisticated analysis employing the NCI-60 panel of tumor cell lines. The intricacies of drug sensitivity, characterized by half-maximal inhibitory concentration (IC50) values, were scrupulously extracted from the comprehensive dataset housed in the CellMiner database (https://discover.nci.nih.gov/cellminer/)^18^. In total, 218 US FDA-approved drugs from the database and the therapeutic effects of 574 different types of drugs or compounds studied in clinical trials were chosen for further analyses. The efficacy of 218 FDA-approved drugs and drugs or compounds from 574 clinical trials was subjected to further study. The “impute”^19^ and “limma” R packages^20^ were employed to investigate the impacts of the expression of FCGBP on the sensitivity of the drug. By leveraging the impute knn function from the impute R package, this study employed a sophisticated approach to estimate missing information pertaining to certain medications. The modeling of drug sensitivity was accomplished through the application of a robust linear model, ensuring a comprehensive exploration of the nuanced relationships within the dataset. Between high- and low-risk FCGBP groups, the difference in the activity of drugs was evaluated via the Wilcoxon method and mapped using the R "ggplot2" and "ggpubr" algorithms.

Furthermore, this investigation systematically assesses the half-maximal inhibitory concentration (IC50) of widely used chemotherapeutic agents sourced from the extensive Genomics of Drug Sensitivity in Cancer (GDSC) library within the context of HNSC^21^. Through the application of the rigorous Wilcoxon rank-sum test, a meticulous comparison was conducted on the IC50 values among cohorts distinguished by elevated and diminished FCGBP expression levels.

### Prediction of candidate miRNAs and lncRNAs

The expression of upstream miRNAs of FCGBP was performed utilizing seven predictive programs, including DIANA TOOLS^22^, miRDB^23^, miRmap^24^, miWalk^25^, RNA22^26^, TargetScan^27^, and microT-CDS^28^. An extensive repository for interaction analysis of miRNAs in TCGA engineering is available at CancerMIRNome (http://bioinfo.jialab-ucr.org/CancerMIRNome/)^29^. Utilizing the CancerMIRNome, datasets of substantially upregulated miRNAs and positive prognostic miRNAs in HNSC were downloaded. The related level between FCGBP and hsa-miR-877-5p and hsa-miR-937-3p was investigated using Pearson correlation. The ENCORI database (https://starbase.sysu.edu.cn/index.php) was employed to predict the upstream candidate lncRNAs of hsa-miR-877-5p^30^. In HNSC, the expression level of lncRNAs and hsa-miR-877-5p or lncRNA–FCGBP were correlated employing Pearson’s correlation coefficient.

### Statistical analysis

The statistical analyses were conducted using R version 3.6.0. Continuous variables were compared between the two groups utilizing the Wilcoxon rank-sum test. Categorical variables underwent comparison using the chi-square test. The prognostic significance of categorical variables was evaluated through the log-rank test. The intricate interplay between FCGBP and the activity of a drug or complex was further elucidated through Spearman’s correlation test. A *p*-value of ≤ 0.05 in all analyses denoted statistically significant differences. Symbols (∗ , ∗  ∗ , ∗  ∗  ∗ , and ∗  ∗  ∗ ∗) were used to indicate *p*-values of ≤ 0.05, < 0.01, < 0.001, and < 0.0001, respectively.

### Ethical approval

Tissue arrays of HNSC were approved by the ethics committee of Fujian Cancer Hospital (Fuzhou, China; numbers K2022-084-01).

## Results

### Pan-cancer analysis of FCGBP expression and its prognostic value

**Figure 1:**
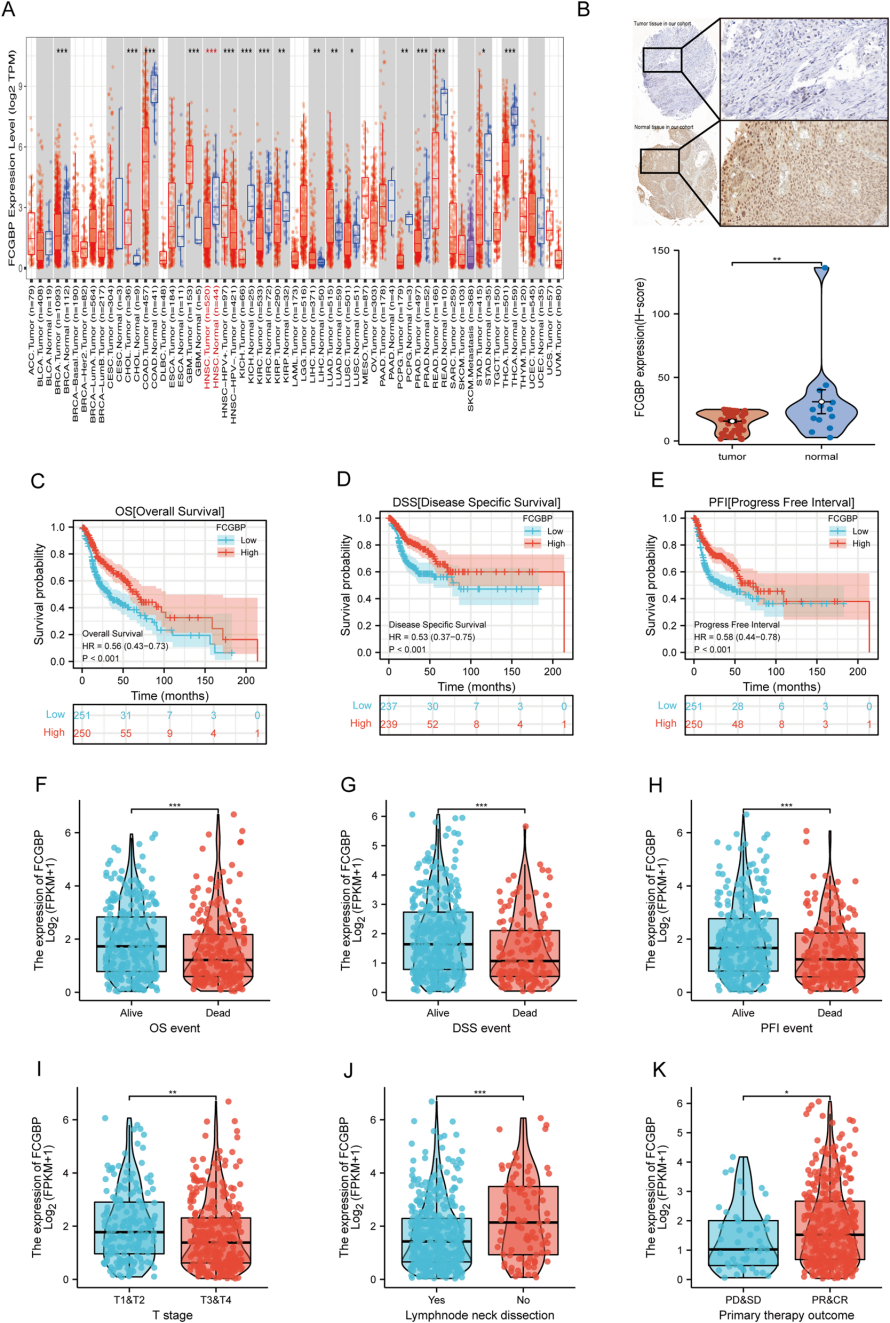
Expression analysis and survival analysis for FCGBP in HNSC. (**A**) The expression of FCGBP in pan-cancer based on TCGA cohort. (**B**) Immunohistochemistry results of FCGBP in cancer and normal tissues. (**C**–**E**) The overall survival (OS), disease specific survival (DSS), Progress Free Interval (PFI) analysis in patients with HNSC. (**F**–**K**) Expression analysis on different OS event (**F**), DSS event (**G**), PFI (**H**), T stage (**I**), lymphnode neck dissection (**J**), primary therapy outcome (**K**).

The first step in exploring the potential features of FCGBP in pan-cancer was to analyze its expression in 22 prevalent types of malignancy. Ten categories of cancer showed significant FCGBP downregulation, namely, BRCA, COAD, HNSC, KICH, LUSC, PCPG, PRAD, READ, STAD, and THCA (Fig. 1A), whereas FCGBP expression level was substantially elevated in CHOL, GBM, LIHC, and LUAD. We then validated the above findings using the GEPIA and UALACAN databases. According to the UALACAN database, the expression of FCGBP was substantially downregulated in twelve types of cancer, including BLCA, BRCA, CESC, COAD, HNSC, KIRC, KIRP, LUSC, PRAD, PCPG, READ, and THCA (Figure S1A). As depicted in Figure S1B, FCGBP expression levels in ACC, HNSC, KICH, LUAD, PAAD, and THYM were downregulated in comparison with those found in the matched normal controls in the GEPIA database. (all *p* < 0.05). Moreover, strong evidence of low FCGBP expression in HNSC tumor tissues was found from our HNSC patient cohorts by immunohistochemistry (Table 1, Fig. 1B). The same findings were further validated in the Human Protein Atlas database (HPA, https://www.proteinatlas.org/, Figure S1C). All the aforementioned results collectively indicate a downregulation of FCGBP expression in HNSCC, suggesting its potential role as a tumor suppressor gene worthy of further exploration.

**Table 1:** Clinicopathological features of the patients in our cohort.

Characteristics	Overall	Characteristics	Overall
Gender, n (%)		Stage, n (%)	
Male	42 (85.7%)	I	4 (8.2%)
Female	7 (14.3%)	II	15 (30.6%)
Age, n (%)		III	11 (22.4%)
< = 50 years old	14	IV	19 (38.8%)
> 50 years old	35	Lymph node metastases, n (%)	
T, n (%)		0	27 (55.1%)
1	5 (10.2%)	1	22 (44.9%)
2	29 (59.2%)	Distant metastases, n (%)	
3	12 (24.5%)	0	48 (98%)
4	3 (6.1%)	1	1 (2%)
N, n (%)		Grade, n (%)	
0	27 (55.1%)	1	20 (44.4%)
1	5 (10.2%)	2	20 (44.4%)
2	17 (34.7%)	3	5 (11.1%)
M, n (%)			
0	48 (98%)		
1	1 (2%)		

For OS (Fig. 1C,F), DSS (Fig. 1D,G), and PFI (Fig. 1E,H), we observed an elevated expression of FCGBP associated with significantly improved prognosis in HNSC patients. In our extensive analysis, the higher expression group demonstrated a strong correlation with a lower clinical T stage (Fig. 1I), absence of lymph node neck dissection (Fig. 1J), and positive treatment response (PR/CR, Fig. 1K) within the TCGA cohort.

**Figure 2:**
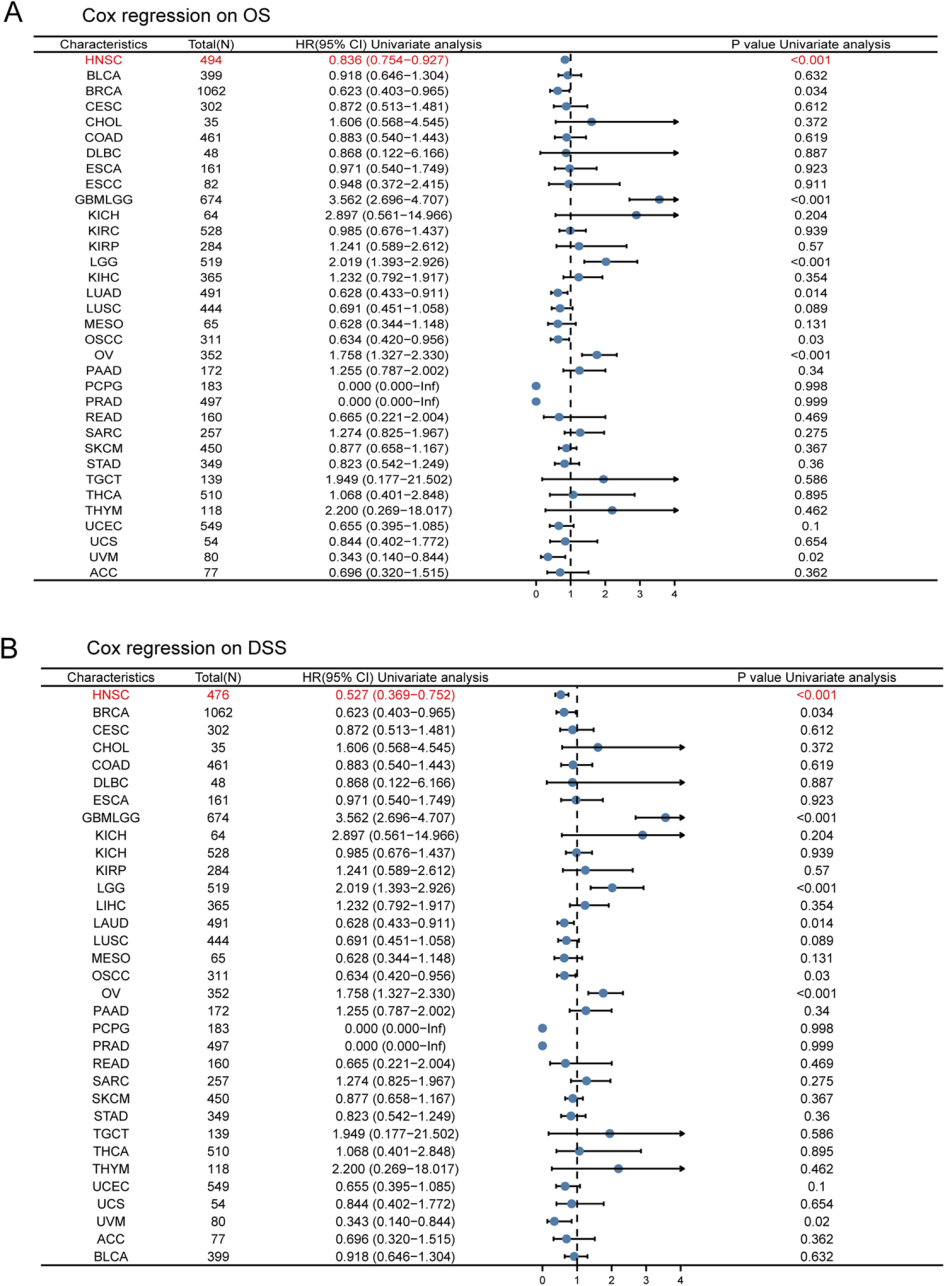
Cox regression analysis on overall survival (**A**) and DSS (**B**) was performed on FCGBP expression in pan-cancer.

Additionally, pan-cancer FCGBP expression was examined using Cox survival analysis. Analyses were performed for both OS and disease-specific survival (DSS). An enhanced FCGBP expression in patients was linked to substantially better prognosis in terms of OS in patients suffering from HNSC, BRCA, GBMLGG, LUAD, OSCC, OV, and UVM (*p* =  < 0.001, 0.034, < 0.001, 0.014, 0.03, < 0.001 and 0.02, respectively). As depicted in Fig. 2A, FCGBP serves as a protective factor of OS in HNSC, BRCA, LAUD, OSCC, and UVM. Meanwhile, Cox regression analysis revealed that an elevated expression level of FCGBP illustrated better prognosis in HNSC, BRCA, LAUD, OSCC, and UVM (*p* =  < 0.001, 0.034, 0.014, 0.03, and 0.02, respectively) for DSS (Fig. 2B). According to these findings, FCGBP expression could be a valuable and favorable prognostic biomarker for those with HNSC.

### The lower FCGBP expression in HNSC is associated with a poorer prognosis, mainly due to the aggregation of tumor-infiltrating macrophages

Head and neck tumors are highly immune-aggressive and severely immunosuppressive diseases^31,32^. Thus, we investigated the association between the degree of HNSC immune infiltration and FCGBP expression.

**Figure 3:**
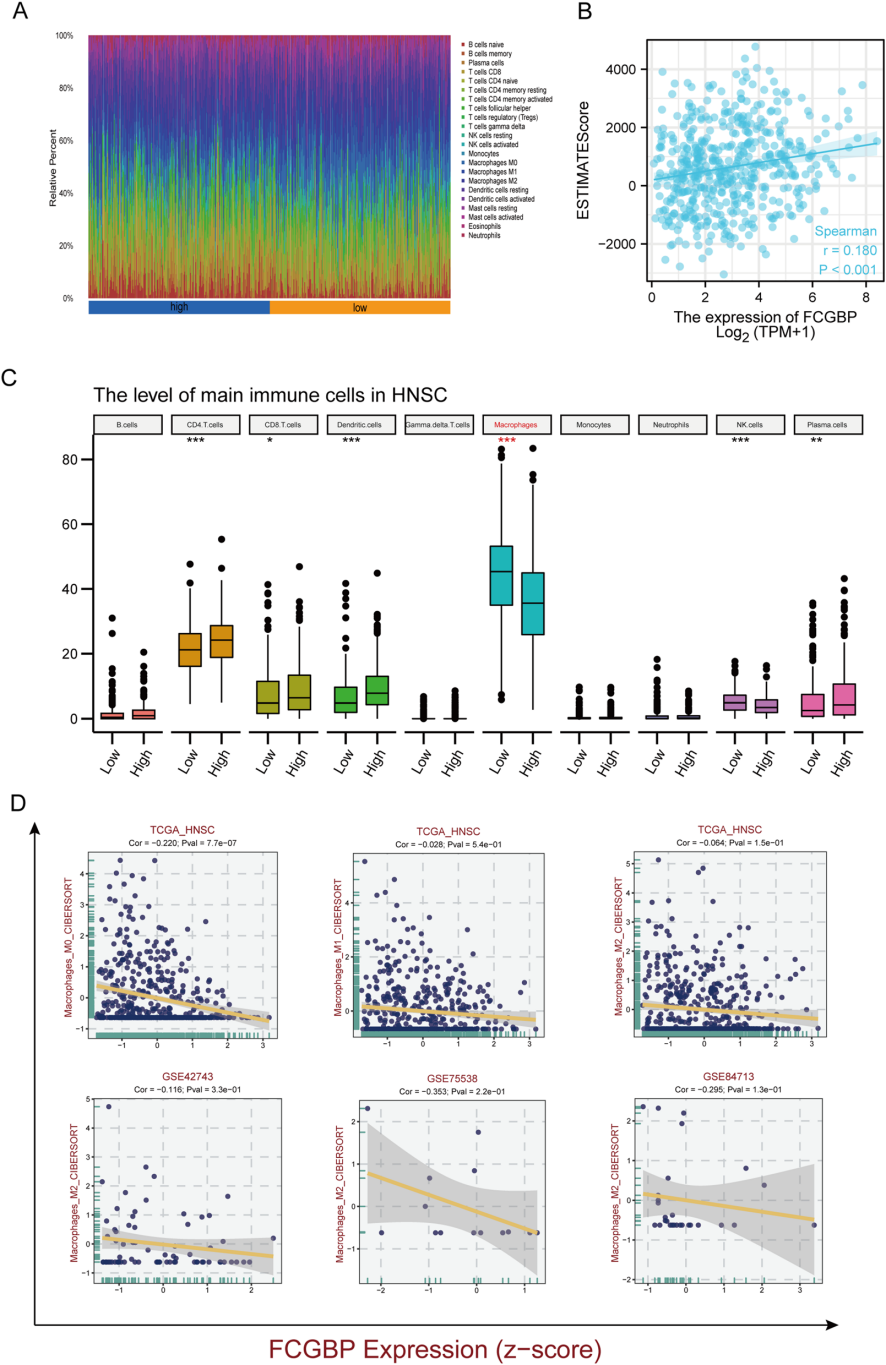
Correlation between FCGBP expression and tumor immune cell infiltration in patients with HNSC. (**A**) The immune landscape of HNSC between high- and low- FCGBP expression group using ssGSEA algorithm. (**B**) Correlation between FCGBP expression and ESTIMATE score. (**C**) HNSC samples were evaluated by CIBERSORT algorithm for tumor-infiltrating immune cells. (**D**) Correlation between FCGBP expression and macrophages in multiple datasets.

In this study, immunograms of 22 different types of HNSCC patients were evaluated using the ssGSEA algorithm (Fig. 3A). FCGBP expression was substantially and positively linked to ESTIMATEscore (Fig. 3B). The above findings were verified employing the CIBERSORT method. As depicted in Fig. 3C, the levels of tumor‐infiltrating CD4 + cells, CD8 + T cells, dendritic cells, macrophages, NK cells, and plasma cells were substantially linked to FCGBP expression (all *p* < 0.05). Furthermore, when we focus on macrophages, it is obvious that the lower FCGBP expression was related to more macrophage infiltration in multiple datasets (Fig. 3D). It was concluded that low levels of the FCGBP gene were associated with a high rate of macrophages.

Further research was done on the correlation between FCGBP expression and biomarkers of M1 or M2 polarized phenotypes of macrophages. FCGBP and the biological markers of M1 and M2 were also discussed in relation to each other.

**Figure 4:**
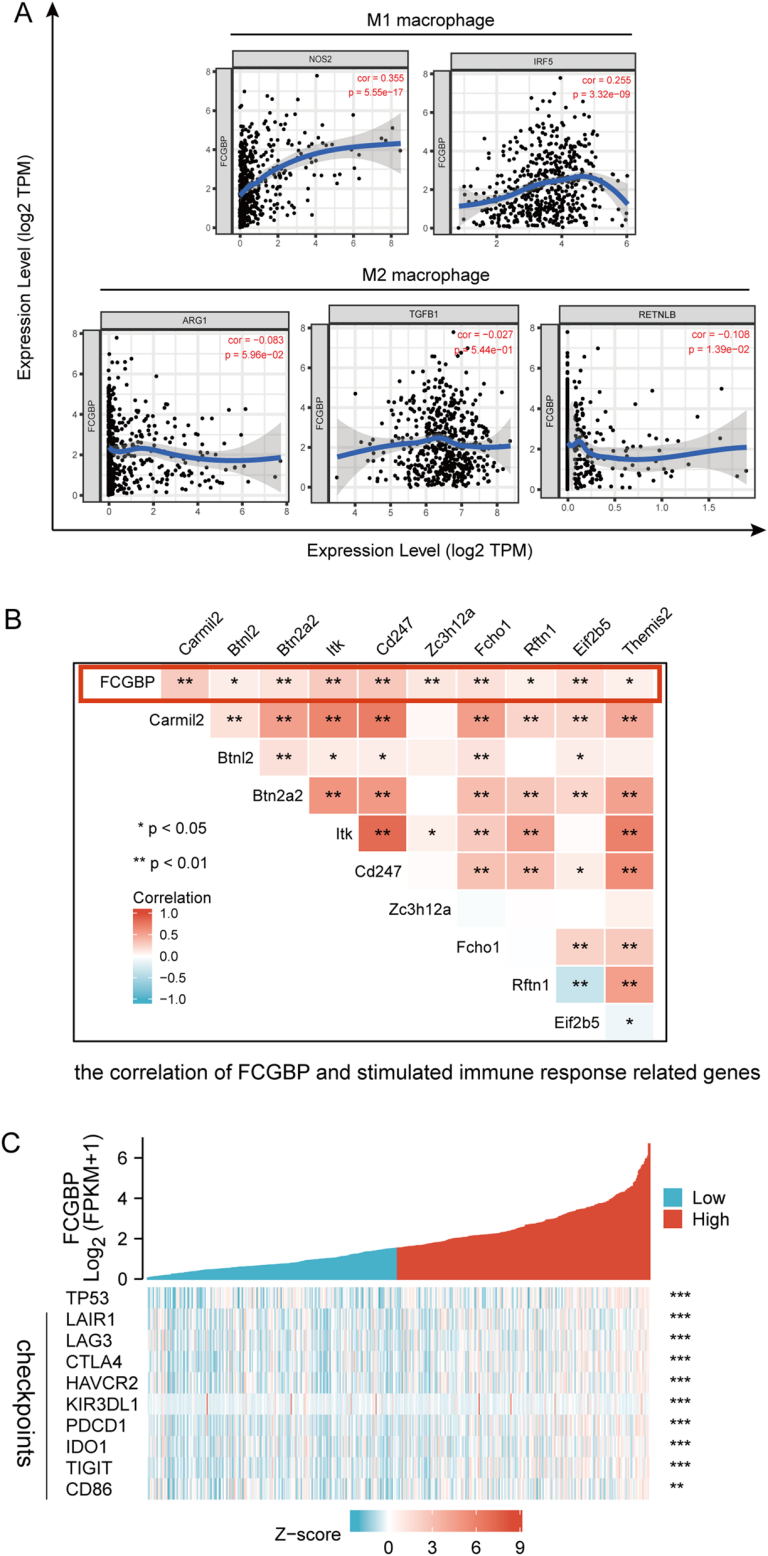
Correlation between FCGBP expression and biomarker of macrophages and immune checkpoints. (**A**) Correlation analysis of FCGBP expression with the markers of M1 or M2 polarized phenotypes of macrophages. (**B**) Correlation of FCGBP and stimulated immune response related genes. (**C**) Correlation of FCGBP and TP53 and immune checkpoints.

The expression of FCGBP and biomarkers of immunosuppressed M2 macrophages was significantly and negatively correlated (RETNLB: r =  − 0.108, *p* < 0.05) but correlated positively with those of M1 macrophages (NOS2: r = 0.355, *p* < 0.001; IRF5: r = 0.255, *p* < 0.001) (Fig. 4A). On the other side, FCGBP expression showed a tightly positive relationship with genes of stimulated immune responses (Fig. 4B). In addition, FCGBP expression was positively related to TP53 and the vast majority of the immune checkpoints, like LAIR, TIGIT, LAG3, HAVCR2, KIR3DL1, PDCD1, IDO1, and CTLA-4 (Fig. 4C).

The data demonstrated that tumor progression and poor prognosis in HNSC patients with low FCGBP expression might partially be linked to macrophages enriched in the tumor immune microenvironment.

### FCGBP expression was an independent risk factor

**Figure 5:**
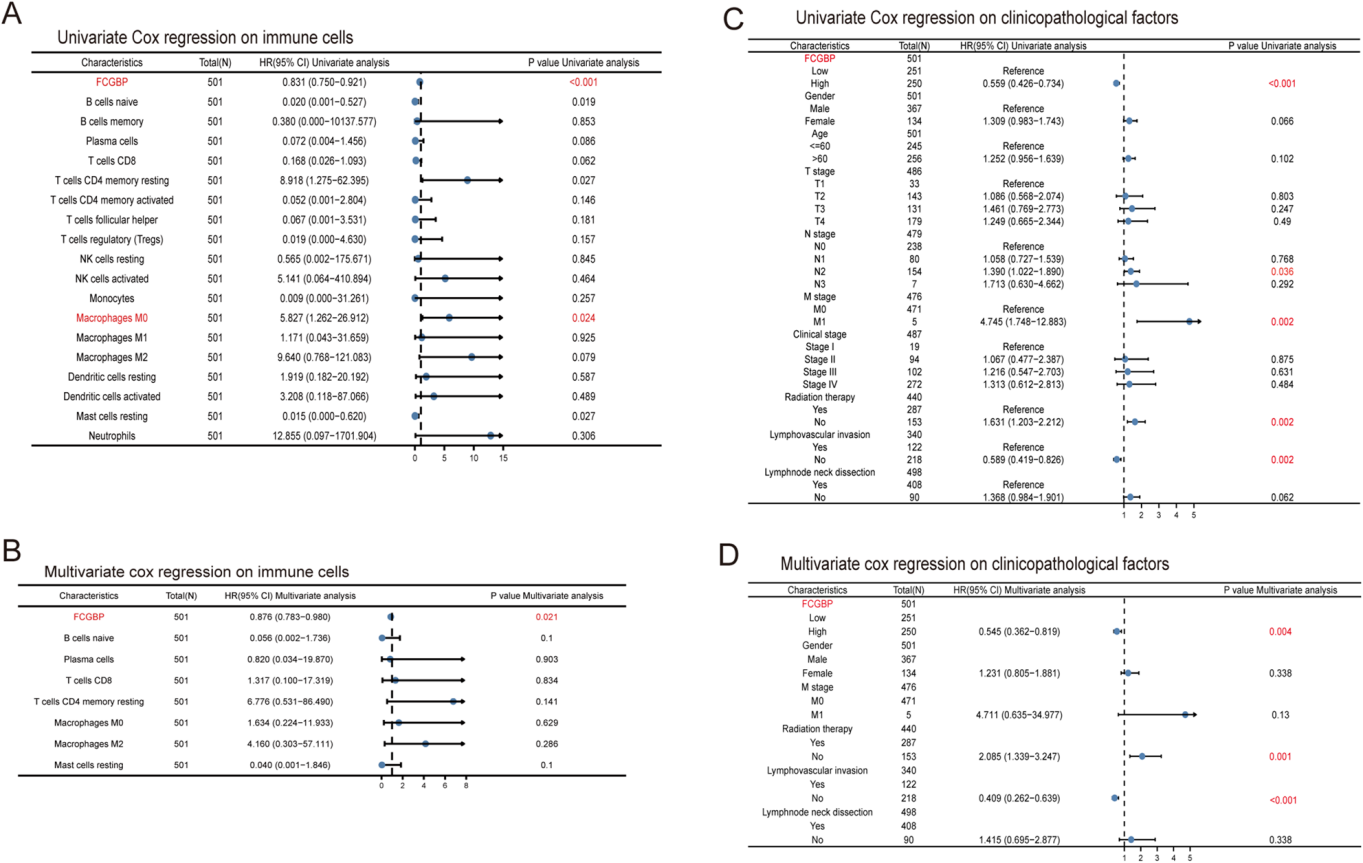
Univariate and multivariate Cox regression analyses on survival and clinicopathological factors in patients with head and neck squamous cell carcinoma (HNSC). (**A**) Univariate Cox regression analysis on survival in HNSC. (**B**) Multivariate Cox regression analysis on survival in HNSC. (**C**) Univariate Cox regression analysis on clinicopathological factors in HNSC. (**D**) Multivariate Cox regression analysis on clinicopathological factors in HNSC.

In Fig. 5A and C, univariate Cox regression analysis discovered that FCGBP expression [hazard ratio (HR): 0.831 (0.750–0.921), *p* < 0.001], macrophages M0 [HR: 5.827 (1.262–26.912), *p* = 0.024], N2 stage [HR: 1.390 (1.022–1.890), *p* = 0.036], M stage [HR: 4.745 (1.748–12.883), *p* = 0.002], radiation therapy [HR: 1.631 (1.203–2.212), *p* = 0.002], and lymphovascular invasion [HR: 0.589 (0.419–0.826), *p* = 0.002] were significant predictors of HNSC patients’ symptoms and treatment outcomes.

The multivariate model included a univariate analysis of variance at *p* < 0.10. Only the following statistics survive multivariate adjustment: FCGBP [HR: 0.876 (0.783–0.980), *p* = 0.021], radiation therapy [HR: 2.085 (1.339–3.247), *p* = 0.001], and lymphovascular invasion [HR: 0.409 (0.262–0.639), *p* < 0.001] as Fig. 5B,D showed.

### Analysis of FCGBP values for drug resistance

**Figure 6:**
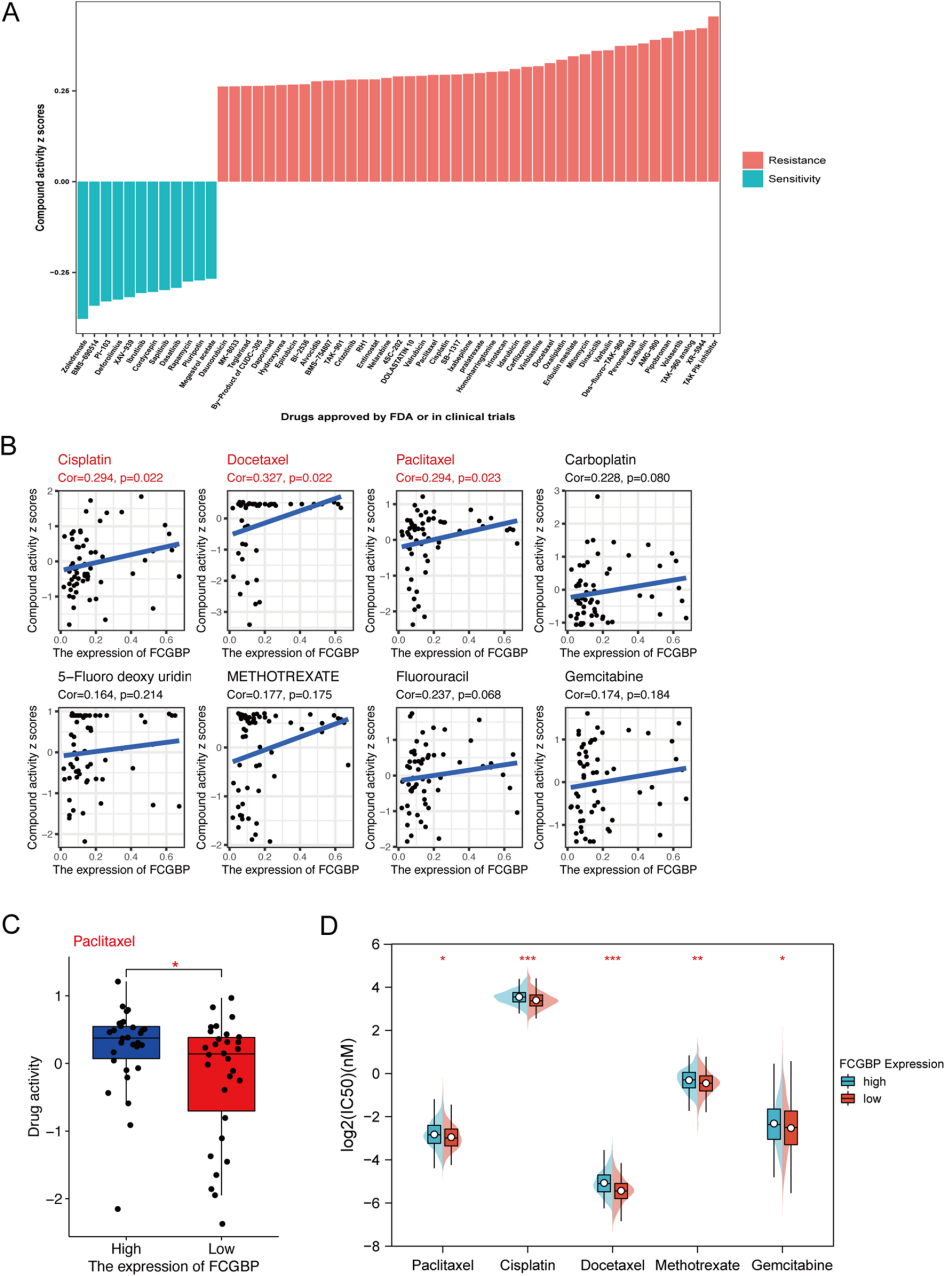
FCGBP expression as a potential predictor for drug sensitivity. (**A**) The compound activity z scores of chosen compounds in relation to FCGBP expression, as shown by correlation analysis. (**B**) Correlation analysis using the Spearman correlation test for FCGBP expression and activity z scores of cisplatin, docetaxel, paclitaxel, carboplatin, 5-fluoro deoxy uridine 10mer, methotrexate, fluorouracil, and gemcitabine. (**C**) Activity z scores of paclitaxel in the high FCGBP expression group compared with those in the low FCGBP expression group. (**D**) IC50 values of chemotherapeutics, including paclitaxel, cisplatin, docetaxel, methotrexate, and gemcitabine in the high FCGBP expression HNSC cell lines compared with those in the low FCGBP expression HNSC patients. HNSC cell lines with low FCGBP expression were found to possess higher IC50 for drugs. The *p*-values were calculated using the Wilcoxon rank-sum test.

In this study, the activity of CellMiner with NCI-60 compounds and RNA-seq data corresponding to NCI-60 were conducted to exploit the value of FCGBP in the prediction of anti-neoplastic drug resistance. It was found that the Compound activity scores were substantially and positively correlated with the sensitivity. We screened 792 drugs or compounds directly related to clinical management from our database, including 218 FDA-approved drugs and 574 drugs of clinical studies. As depicted in Fig. 6A, the results discovered that expression of the FCGBP gene was related to increased survivability of 12 medicines or combinations and the reduced activity of 43 drugs or compounds.

As per the National Comprehensive Cancer Network (NCCN), the most popular chemotherapy agents used to treat head and neck tumors include cisplatin, methylpteridine paclitaxel, docetaxel, 5-FU, carboplatin, and gemcitabine. As depicted in Fig. 6B, a positive correlation was discovered between the expression of FCGBP and the potency of cisplatin, docetaxel, and paclitaxel (r = 0.294, *p* = 0.022, r = 0.327, *p* = 0.022, and r = 0.294, *p* = 0.023, respectively;). The effectiveness of carboplatin, methotrexate, 5-FU, or gemcitabine has also been shown to be positively correlated with FCGBP (all *p* > 0.05).

To determine if FCGBP expression influences drug activity, we also classified patients into categories with high or low FCGBP expression levels based on the median value. As depicted in Fig. 6D, the findings demonstrated that patients with low FCGBP expression had much less paclitaxel activity (*p* < 0.05). We classified the FCGBP expression values in the HNSC cell lines into high and low categories to assess if FCGBP expression influenced the drug activity in HNSC. Higher IC50 values for paclitaxel, cisplatin, docetaxel, methotrexate, and gemcitabine (*p* < 0.05, < 0.001, < 0.001, < 0.01, < 0.05, respectively; Fig. 6D) were found for patients with low expression of FCGBP.

Accordingly, FCGBP expression may serve as a predictor of paclitaxel resistance, which might contribute to poor prognosis in HNSC.

### Prediction regulatory miRNAs of FCGBP

Research has shown that non-coding RNAs can regulate gene expression. MiRNAs (microRNAs) bind to target mRNAs to perform their function^33^.

**Figure 7:**
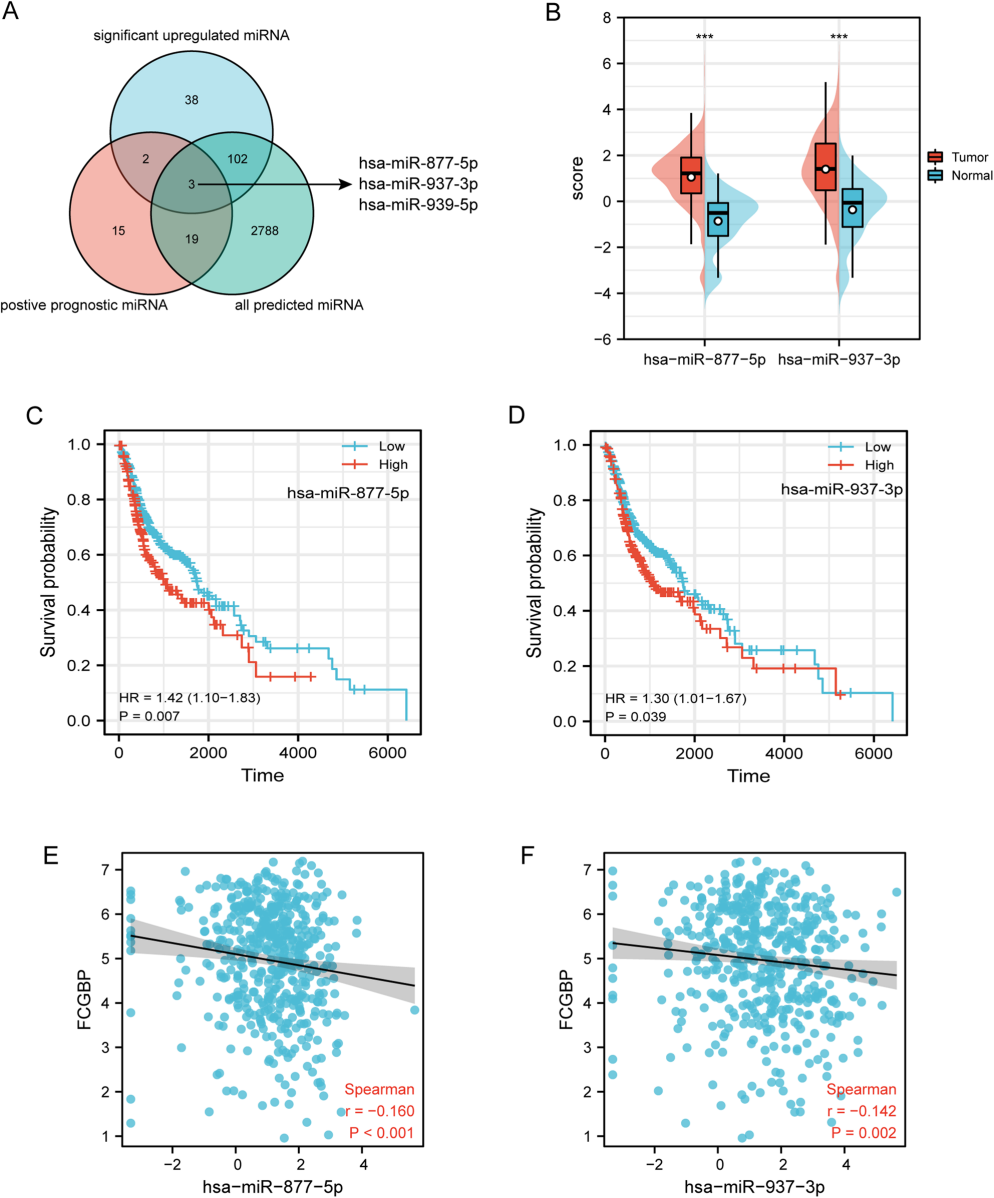
Identification of hsa-miR-877-5p and hsa-miR-937-3p as potential upstream miRNAs of FCGBP in HNSC. (**A**) Venn diagram of predicted miRNAs, upregulatedmiRNAs, and miRNAs related to better survival in HNSC. (**B**) The expression of hsa-miR-877-5p and hsa-miR-937-3p in HNSC and control normal samples using the CancerMIRNome database. (**C**–**D**) The prognostic value of hsa-miR-877-5p (**C**) and hsa-miR-937-3p (**D**) in HNSC. (**E**–**F**) The expression correlation between FCGBP and hsa-miR-877-5p (**E**) and hsa-miR-937-3p (**F**) in HNSC.

In order to clarify how ncRNAs may regulate FCGBP, we first predicted miRNAs binding to FCGBP. Employing seven prediction methods, 2912 miRNAs have been identified (Supplementary Material Table S1). In terms of the mechanism of miRNA-target action, the interaction between upstream miRNAs and FCGBP may lead to a decreased trend. As depicted in Fig. 7A, in the CancerMIRNome analysis of the TCGA database, 39 positive prognostic miRNAs and 145 substantially upregulated miRNAs were identified in HNSC.

After excluding hsa-miR-939-50 which could not be found in NCBI datasets, only two miRNAs, hsa-miR-877-5p and hsa-miR-937-3p, met all these requirements. hsa-miR-877-5p and hsa-miR-937-3p were significantly upregulated in HNSC, and their downregulation was linked to superior prognosis of HNSC patients (Fig. 7B,C,D, *p *< 0.05). FCGBP expression was discovered to be substantially and negatively linked to the expression of hsa-miR-877-5p and hsa-miR-937-3p (r = -0.16, *p* < 0.001; r = -0.142, *p* = 0.002), according to Pearson’s correlation analysis (Fig. 7E,F). Taken together, hsa-miR-877-5p and hsa-miR-937-3p might be upstream miRNAs of FCGBP in HNSC.

### Prediction of upstream lncRNAs of hsa-miR-877-5p

**Figure 8:**
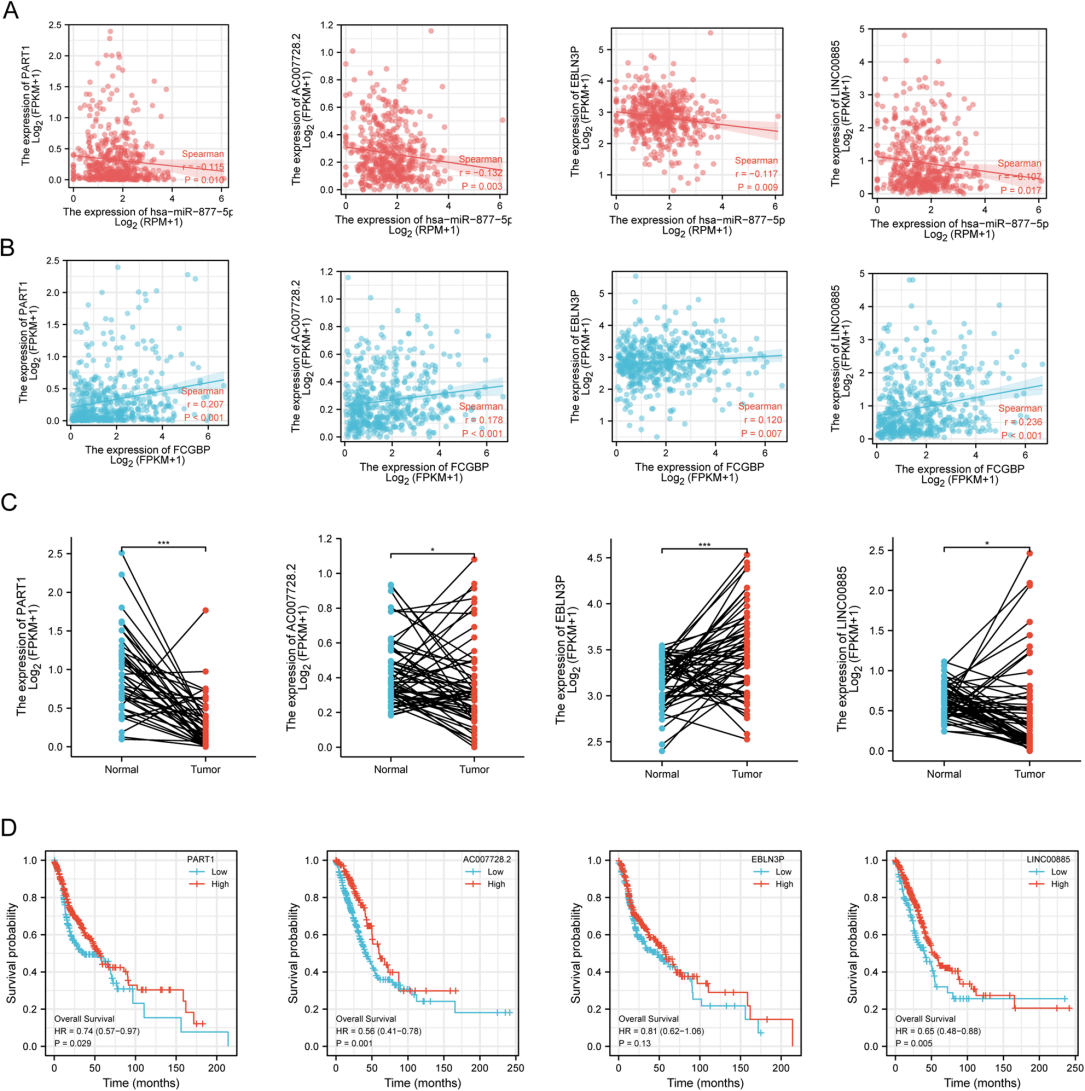
Correlation analysis, expression analysis, and survival analysis for upstream lncRNAs of hsa-miR-877-5p in HNSC. (**A**) Correlation analysis between hsa-miR-877-5p and PART1, AC007728.2, EBLN3P, and LINC00885, respectively. (**B**) Correlation analysis between FCGBP and PART1, AC007728.2, EBLN3P, and LINC00885. (**C**) The expression of PART1, AC007728.2, EBLN3P, and LINC00885 in TCGA HNSC compared with that in normal tissues. (**D**) The OS analysis for PART1, AC007728.2, EBLN3P, and LINC00885 in HNSC.

The upstream lncRNAs of hsa-miR-877-5p and hsa-miR-937-3p were also predicted employing the ENCORI database. We eliminated hsa-miR-937-3p due to no results for the search in ENCORI database. Up to 35 potential lncRNAs targeting hsa-miR-877-5p were predicted. The results reveal a substantial negative correlation between the gene levels of the four lncRNAs and the hsa-miR-877-5p gene in HNSC cells as well as positively linked to the expression of FCGBP (Fig. 8A,B), including PART1 (ENSG00000152931), AC007728.2 (ENSG00000261644), EBLN3P (ENSG00000281649), and LINC00885 (ENSG00000224652), In this way, the above lncRNAs are associated with the mechanism of competing endogenous RNA (ceRNA). Next, we detected the four associated lncRNAs expression by TCGA cohort. As suggested in Fig. 8C, PART1, AC007728.2, and LINC00885 were significantly downregulated in HNSC in comparison with normal controls. We then evaluated the ability of the four lncRNAs to predict HNSC prognostic outcomes. Patients with HNSCs with an elevated expression level of PART1, AC007728.2, and LINC00885 were substantially related to better OS (*p* = 0.029, 0.001, 0.005, respectively; Fig. 8D).

Based on the correlation, expression, and survival analysis results, PART1, AC007728.2, and LINC00885 were considered to be the most possible upstream lncRNAs of the hsa-miR-877-5p/FCGBP axis in HNSC.

## Discussion

In this study, analysis of FCGBP gene expression and survival showed that the clinical outcome was poor in HNSC patients with low FCGBP expression levels. Correlation and intra-group comparison studies of different FCGBP expression groups exhibited that FCGBP might act as paclitaxel, cisplatin, and docetaxel resistance predictor and was linked to abundant infiltration of macrophages and immune checkpoints associated with tumorigenic.

FCGBP is a crucial component of the human mucosa defense and is involved in the immune protection of the body and the inflammatory response in the intestine^34^. It is reported that FCGBP has an essential impact on the onset, progression, and prognosis of several malignant disorders^13,40^.

FCGBP alternative splicing and FCGBP mutations may be relevant to the mechanism of lung carcinogenesis^9^. FCGBP at transcriptional levels was significantly reduced in prostate adenocarcinoma tissue from humans and transgenic mice^11^. In addition, FCGBP protein has an independent prognostic profile in gallbladder adenocarcinoma and metastatic colorectal cancer^14^. In other words, FCGBP is involved in the interaction of goblet cellular products with bacterially triggered metabolic compounds^35^. As tumors progress, metabolic demands change and new dependencies arise with tumor treatment and metastasis^36^.

In alignment with the established HNSC guidelines, conventional chemotherapy, notably utilizing paclitaxel and cisplatin, stands as a cornerstone in treatment strategies^1^. However, it is imperative to acknowledge the potential challenges posed by typical chemotherapy regimens, which may inadvertently impact the intricate balance of the intestinal microbiota, leading to adverse effects and diminished therapeutic efficacy^37^. Our study provides valuable insights into the intricate relationship between FCGBP downregulation and paclitaxel resistance. Within the HNSC microenvironment, the administration of paclitaxel via intravenous routes appears to influence mucosal immunity, potentially contributing to the development of resistance to chemotherapy. This observed effect could be linked to a plausible mechanism involving the reduction in the number of gut-associated lymphoid tissue cells^38^. This finding sheds light on the multifaceted consequences of paclitaxel administration beyond its direct cytotoxic effects, emphasizing the importance of understanding and mitigating potential immunomodulatory impacts in HNSC treatment.

Furthermore, the efficiency of cisplatin chemotherapy emerges as an area ripe for intervention through the regulation of the intestinal microenvironment^39^. The intricate interplay between platinum-sensitive and platinum-insensitive patients, as evidenced by significant differences in FCGBP expression, underscores the dynamic nature of mucosal immune function in the context of HNSC chemotherapy response^15^. These studies suggest that HNSC patients are more tolerant to chemotherapy, possibly due to changes in their mucosal immune function.

By analyzing data from patients with head and neck tumors, we discovered that FCGBP expression was linked to paclitaxel resistance. Additionally, there was a tendency regarding the link to docetaxel and cisplatin resistance. Here, we found from the data of patients with HNSC that FCGBP expression was linked to resistance to paclitaxel. Also, the resistance to cisplatin and docetaxel presents a trend as well. More advanced studies are awaited to elucidate the relationship of resistance to paclitaxel and docetaxel in HNSC and mucosal immune.

In the current world of immunotherapy, the clinical protocol for HNSC has changed considerably. HNSC is a type of malignancy with suppressed immune surveillance mechanisms^31^. The outcomes of this work exhibited a substantial link between FCGBP and M2 macrophages in the tumor microenvironment.

FCGBP functions in the mucosal epithelial cell with a natural immune function, which regulates the adhesion of mucosal epithelial cells and thereby contributes to the onset and advancement of disease^40^. Possibly, this is why FCGBP is associated with the tumor microenvironment.

Secondly, FCGBP-interacted TFF2, a constituent of the innate immune defense of the mucosal epithelium in the intestine with the help of immunoglobulins on mucosal surfaces supports the inflammatory response in macrophages^41^. Previous experiments have shown that FCGBP plays an active part in immunological defense in the intestine^42^. Low FCGBP expression in HNSC contributes to the deficiency of mucosal immune defense, leading to an immunosuppressive microenvironment^8^.

Widespread investigations have highlighted ncRNAs, including miRNAs, and circRNAs, which can exert a regulatory role on gene expression. Numerous ncRNAs act as competitive endogenous RNAs (ceRNAs) via the mechanism of regulating the biological properties of tumors^33,43^. To elucidate the regulatory mechanism of ceRNA and FCGBP, we have used a range of predictive software to analyze FCGBP and potential lncRNAs.

The PART1/AC007728.2/LINC00885/hsa-miR-877-5p/FCGBP axis was identified as a potential regulatory pathway in HNSC. PART1, AC007728.2, and LINC00885 expression levels were elevated and substantially linked to OS in HNSC patients, which was in line with the role of PART1 in lung squamous cell carcinoma^44^ and the role of LINC00885 in breast cancer^45^. Correspondingly, hsa-miR-877-5p was upregulated and was substantially linked to a worse prognosis of HNSC from our findings, which fit with early research in LUAD^46^.

In summary, the discovery of FCGBP as a potential predictor for drug resistance, especially against paclitaxel, cisplatin, and docetaxel, offers promising prospects for personalized treatment in HNSC patients. Its correlation with low expression and unfavorable clinical outcomes suggests it could serve as a valuable prognostic biomarker. Furthermore, the association between FCGBP and immune infiltration patterns indicates its role in predicting responses to immunotherapies. Exploring its interplay with immune checkpoints and tumor-associated macrophages lays the groundwork for targeted immunotherapies. Clinical trials combining immunomodulatory agents with FCGBP analysis may improve treatment responses.

In comparison to previous studies, our study has some shortcomings^47–49^. Firstly, it differs from one type of head and neck tumor to another. Sub-location and different management practices have a great impact on the prognosis of the patient and may be biased. Secondly, retrospective analyses are susceptible to selection bias, as patient data are collected based on historical records and may not capture all relevant variables. Thirdly, the upstream axis of FCGBP and the types of invasive immune cells were estimated. Estimation errors may have occurred. A large number of trials and multicentre prospective trials are essential to better substantiate the therapeutic effects and possible mechanisms of FCGBP.

## Conclusion

**Figure 9:**
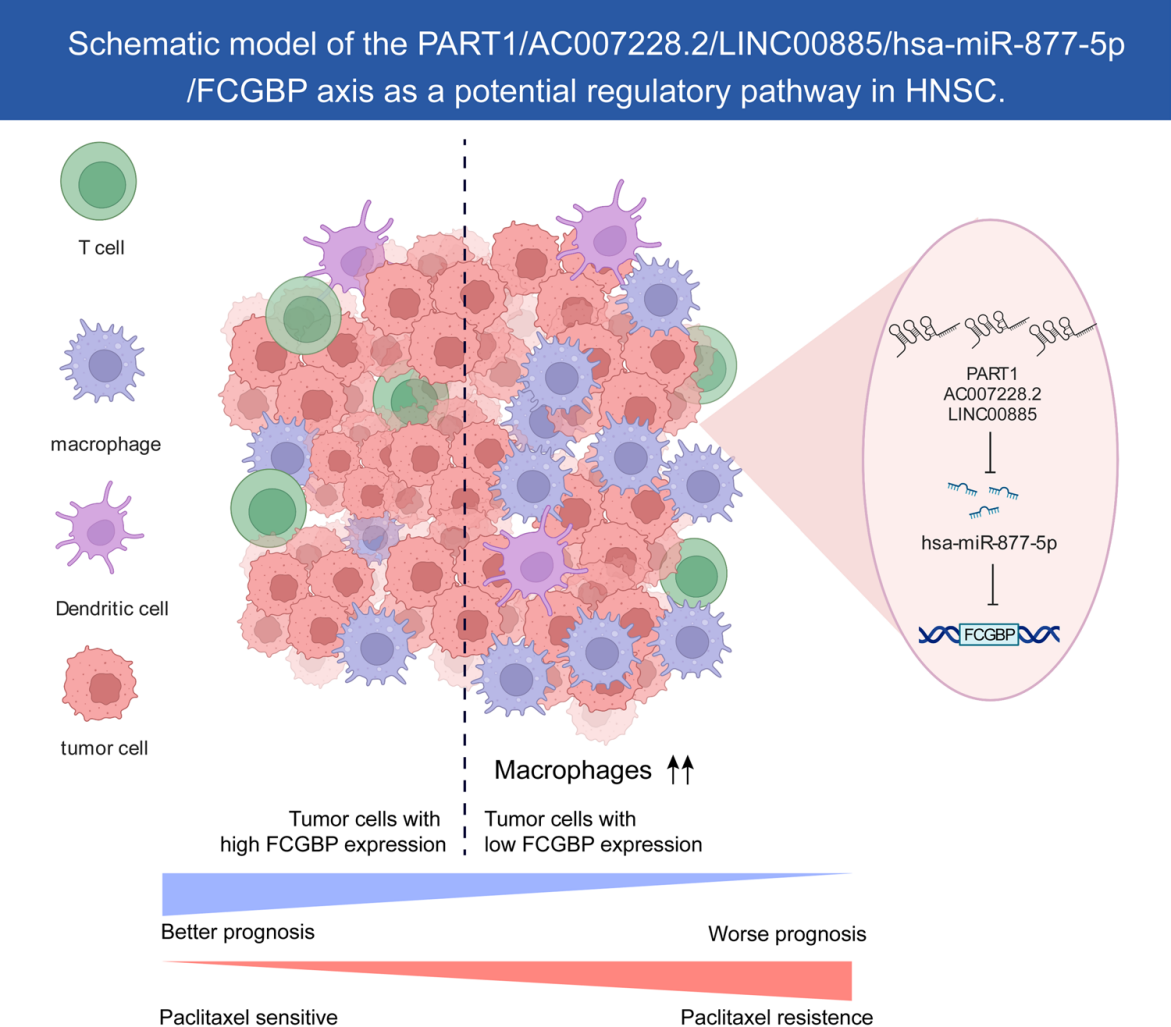
Schematic model of the PART1/AC007728.2/LINC00885/hsa-miR-877-5p/ FCGBP axis as a prospective regulatory pathway in HNSC.

This study demonstrates that low expression levels of the FCGBP are associated with the prognosis of HNSC. As depicted in Fig. 9, we identified the PART1/AC007728.2/LINC00885/hsa-miR-877-5p/FCGBP axis as a prospective regulatory pathway in HNSC. In general, the low level of FCGBP expression may lead to a certain level of paclitaxel resistance in HNSC cells and enhance their immunosuppressive capacity against anti-tumor cells. However, more groundwork and experiments are yet to be done to confirm these findings.

### Supplementary Information

Supplementary Figure S1.

Supplementary Table S1.

## Data Availability

The public dataset used in this study is freely available at https://xenabrowser.net/, https://www.ncbi.nlm.nih.gov/, and https://pdc.cancer.gov/pdc/browse.
