# Supplementary material for: Non-coding RNA-related FCGBP downregulation in head and neck squamous cell carcinoma: a novel biomarker for predicting paclitaxel resistance and immunosuppressive microenvironment
Source: Sci Rep. 2024 Feb 23;14:4426. doi: 10.1038/s41598-024-55210-6 (PMC10891054; doi:10.1038/s41598-024-55210-6)
Supplement: Supplementary file 1 — Supplementary Figure S1. [file 41598_2024_55210_MOESM1_ESM.pdf]

Non-coding RNA-related FCGBP downregulation in head and neck squamous cell carcinoma: A novel biomarker for predicting paclitaxel resistance and immunosuppressive microenvironment

Qin Ding[1,2†], Fengjie Lin[1,2†], Zongwei Huang[1,2†], Ying Li[1,2], Sunqin Cai[1,2], Xin Chen[1,2], Hui Liu[1,2*], Sufang Qiu[1,2*]

[1]Department of Radiation Oncology, Clinical Oncology School of Fujian Medical University, Fujian Cancer Hospital, Fuzhou, China

[2]Fujian Provincial Key Laboratory of Translational Cancer Medicine, Fuzhou, China

[*]Correspondence: Sufang Qiu, sufangqiu@fjmu.edu.cn, Department of Radiation Oncology, Clinical Oncology School of Fujian Medical University, Fujian Cancer Hospital, Fuzhou, 350014, China

Hui Liu, liuhuifj@sina.com, Department of Radiation Oncology, Clinical Oncology School of Fujian Medical University, Fujian Cancer Hospital, Fuzhou, 350014, China

[†]These authors have contributed equally to this work.

Supplementary Materials:

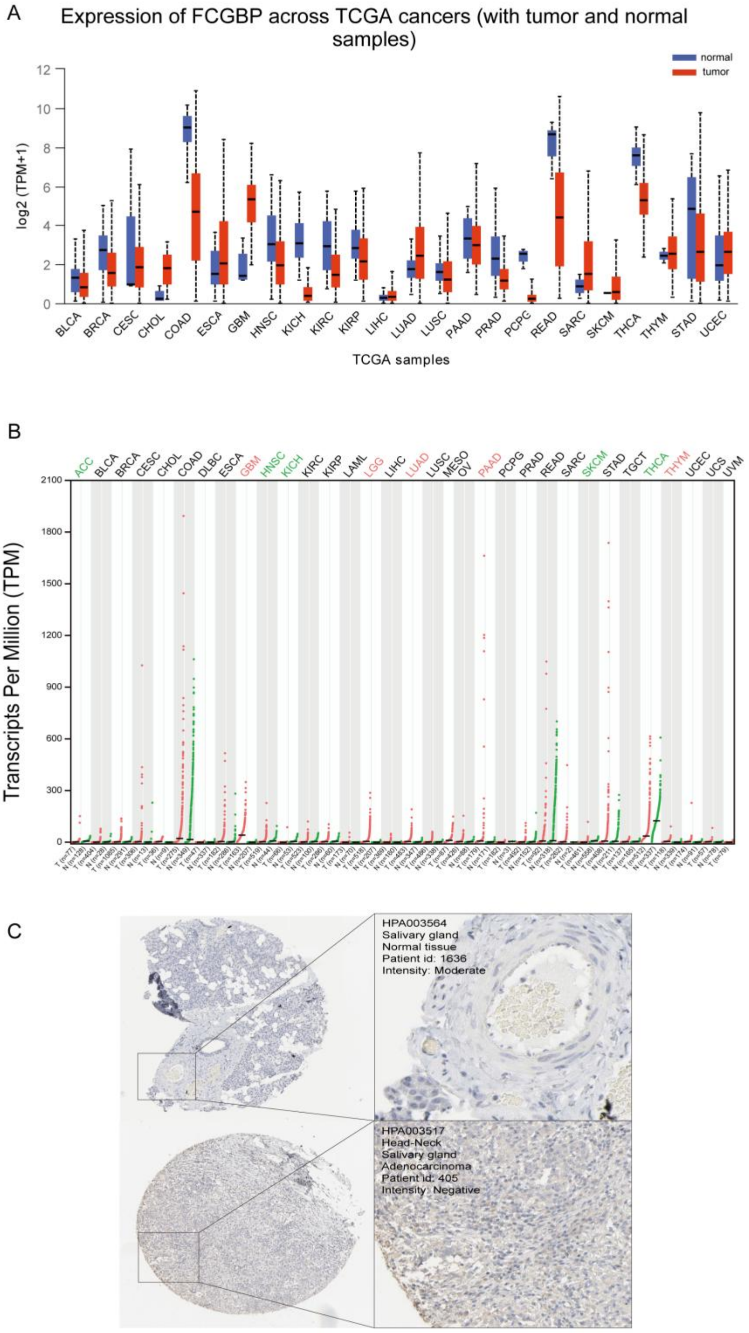

Figure S1: Expression analysis and survival analysis for FCGBP in pan-cancer by UALCAN and GEPIA. (A) The expression of FCGBP in pan-cancer based on TCGA cancer and normal data analyzed using UALCAN. (B) The expression of FCGBP in pan-cancer based on TCGA cancer and normal data analyzed using UALCAN using

the GEPIA database. *p value < 0.05; **p value < 0.01; ***p value < 0.001. (C) Immunohistochemistry results of FCGBP in cancer and normal tissues from the Human Protein Atlas database.

Table S1: Predicted miRNAs by seven prediction programs.
